# Association between Serum 25-hydroxyvitamin D Levels and Incidences of Infection in Long-Term Care

**DOI:** 10.1093/geroni/igab046.2534

**Published:** 2021-12-17

**Authors:** Ronna Robbins, Monica Serra, Margaret Briley

**Affiliations:** 1 Audie Murphy VA Medical Center, San Antonio, Texas, United States; 2 UT Health San Antonio - Barshop Institute, San Antonio, Texas, United States; 3 University of Texas at Austin, Austin, Texas, United States

## Abstract

Insufficient serum 25-hydroxyvitamin D (25(OH)D) concentrations are associated with increased respiratory tract infections, influenza, and other infectious diseases. As the world deals with the COVID-19 pandemic, the interest of adequate serum levels to reduce the risk of infection has surfaced. This study determined if the number of infections per year are associated with serum 25(OH)D concentrations in long-term care (LTC). Participants (≥ 65 years) in a cross-sectional study were recruited across five LTC communities in Texas. Medical records were used to collect a one-year medical history using double-blind protocols. Blood draws were collected to measure serum 25(OH)D concentrations. Medical records were used to classify infections based upon documentation of signs and symptoms of infection concurrent with either a physician’s note or antibiotic/antiviral medication prescription. Race, BMI, sex, age, and liver and renal disease diagnoses were used as confounders. Of the 177 participants (89% Caucasian, 63% female, mean age 83 years) 69% had ≥1 infection over year and 55% had insufficient serum 25(OH)D concentrations <30 mg/mL (mean 32.6 ng/mL). Linear regression did not show a significant association between serum 25(OH)D concentrations and number of infections (β 0.003; 95% CI -0.014, 0.018; p=0.760). Additionally, insufficient serum concentration did not increase the odds of having an infection (OR 1.02; 95% CI 0.05, -19.34; p=0.987). This study did not show a significant association between infection rates and serum 25(OH)D concentrations. However, further research is needed to determine if vitamin D supplementation could be an effective therapeutic intervention to reduce infection rates, including COVID-19.

